# Acrolein scavenger dimercaprol offers neuroprotection in an animal model of Parkinson’s disease: implication of acrolein and TRPA1

**DOI:** 10.1186/s40035-021-00239-0

**Published:** 2021-04-28

**Authors:** Liangqin Shi, Yazhou Lin, Yucheng Jiao, Seth A. Herr, Jonathan Tang, Edmond Rogers, Zhengli Chen, Riyi Shi

**Affiliations:** 1 College of Basic Medicine, Chengdu University of Traditional Chinese Medicine, Chengdu 610075, China; 2 Center for Paralysis Research & Department of Basic Medical Sciences, College of Veterinary Medicine, Purdue University, West Lafayette, IN 47907 USA; 3 Laboratory of Animal Disease Model, College of Veterinary Medicine, Sichuan Agricultural University, Chengdu, 625014 China; 4 Department of Orthopedics, Ruijin Hospital, School of Medicine, Shanghai Jiao Tong University, Institute of Trauma and Orthopedics, Shanghai, 200025 China; 5 Weldon School of Biomedical Engineering, Purdue University West Lafayette, West Lafayette, IN 47907 USA

**Keywords:** Oxidative stress, Parkinson’s disease, Neuroinflammation, Acrolein, TRPA1, Dimercaprol

## Abstract

**Background:**

The mechanisms underlying lesions of dopaminergic (DA) neurons, an essential pathology of Parkinson’s disease (PD), are largely unknown, although oxidative stress is recognized as a key factor. We have previously shown that the pro-oxidative aldehyde acrolein is a critical factor in PD pathology, and that acrolein scavenger hydralazine can reduce the elevated acrolein, mitigate DA neuron death, and alleviate motor deficits in a 6-hydroxydopamine (6-OHDA) rat model. As such, we hypothesize that a structurally distinct acrolein scavenger, dimercaprol (DP), can also offer neuroprotection and behavioral benefits.

**Methods:**

DP was used to lower the elevated levels of acrolein in the basal ganglia of 6-OHDA rats. The acrolein levels and related pathologies were measured by immunohistochemistry. Locomotor and behavioral effects of 6-OHDA injections and DP treatment were examined using the open field test and rotarod test. Pain was assessed using mechanical allodynia, cold hypersensitivity, and plantar tests. Finally, the effects of DP were assessed in vitro on SK-N-SH dopaminergic cells exposed to acrolein.

**Results:**

DP reduced acrolein and reversed the upregulation of pain-sensing transient receptor potential ankyrin 1 (TRPA1) channels in the substantia nigra, striatum, and cortex. DP also mitigated both motor and sensory deficits typical of PD. In addition, DP lowered acrolein and protected DA-like cells in vitro. Acrolein’s ability to upregulate TRPA1 was also verified in vitro using cell lines.

**Conclusions:**

These results further elucidated the acrolein-mediated pathogenesis and reinforced the critical role of acrolein in PD while providing strong arguments for anti-acrolein treatments as a novel and feasible strategy to combat neurodegeneration in PD. Considering the extensive involvement of acrolein in various nervous system illnesses and beyond, anti-acrolein strategies may have wide applications and broad impacts on human health.

**Supplementary Information:**

The online version contains supplementary material available at 10.1186/s40035-021-00239-0.

## Background

Parkinson’s disease (PD) is a common chronic neurodegenerative disease characterized as a severe movement disorder [1–3]. The critical pathology of PD is the progressive death of dopaminergic (DA) neurons in the substantia nigra (SN) that project into the striatum, a brain area critical for motor function [4–7]. Despite decades of research efforts, the mechanisms of DA neuron destruction are largely unknown. Consequently, no established treatment is available to curtail the neuronal cell death and the pathological progression of PD, and the only available clinical therapeutic options are mainly for symptom relief [3, 8]. This represents a significant, unmet medical need and warrants continuous major efforts to decipher the mechanisms and devise strategies that can deter the deterioration of DA neurons.

While the exact cause is unknown, oxidative stress has been postulated as one of the most important contributors to nigral cell death in PD [9–11]. To this end, we have previously reported that toxic aldehydes, such as acrolein, may play a key role in PD pathology [11, 12]. Acrolein, as both a product of and catalyst for lipid peroxidation, is a key factor in perpetuating oxidative stress [13–18]. More specifically, we have demonstrated that acrolein is elevated in a rat model (6-hydroxydopamine [6-OHDA]) of PD [11]. Furthermore, while injecting acrolein into a rat brain can reproduce PD-like symptoms and pathologies mirroring those seen in 6-OHDA-injected rats, lowering acrolein using the scavenger hydralazine (HZ) mitigates PD pathologies and motor deficits [11, 19]. Finally, the most direct evidence for a specific role of acrolein in PD may be that acrolein can lead to α-synuclein (α-syn) aggregation, a hallmark of PD pathology, in both cell-free systems and in vivo experiments [11, 12]. Therefore, acrolein may be a key therapeutic target, and lowering acrolein may represent a novel neuroprotective strategy for mitigating DA cell death. In fact, we have already shown that the acrolein scavenger HZ has neuroprotective effects by lowering acrolein in 6-OHDA-injected rats [11].

However, while HZ has been shown to reduce acrolein, potentially offering neuroprotection in PD, it has other effects that are not related to aldehyde-scavenging [20–22]. Specifically, HZ is an FDA-approved medication for hypertension, and the side effect of lowering blood pressure makes it challenging to justify its use in PD patients [23–28]. Therefore, other aldehyde scavengers with less concerning side effects, while also being able to effectively scavenge acrolein, will have a better chance for use in patients.

To achieve this goal, in this study, we set out to test the neuroprotective effects of dimercaprol (DP), another known acrolein scavenger, in PD [29]. DP is also an FDA-approved medication, indicating safe use in humans. Unlike HZ, DP does not have a side effect of lowering blood pressure. In addition to testing the effect of DP on mitigating PD-related motor deficits, we also tested the ability of DP to alleviate hypersensitivity, a known symptom of PD [30–33]. This hypothesis is based on the evidence that acrolein can elicit neuropathic pain, such as that in spinal cord injury (SCI) [17, 20, 34–38]. Furthermore, we examined the expression of transient receptor potential ankyrin 1 (TRPA1) channel. TRPA1 is known to be activated and upregulated by acrolein [37, 39]. Research has shown that TRPA1 is an important mediator of hyperalgesia, with relevance to pain and aldehydes [40]. Therefore, we examined the change of TRPA1 in PD-related areas where acrolein is known to be elevated: SN and striatum, as well as in cortex.

## Materials and methods

### Animals

Male Sprague–Dawley rats (Harlan Laboratory, Indianapolis, IN) weighing approximately 250 g at the beginning of the experiment were used in this study. Animals were housed under conditions of controlled temperature (25 °C) and illumination (12 h light; 12 h dark) and were given free access to standard diet and water. All experimental procedures were approved by the Purdue University Animal Care and Use Committee (protocol # 1903001867) and followed the ARRIVE guideline (Animal Research: Reporting of In Vivo Experiments). Before the experiment, all animals were housed at least 1 week to allow for acclimation to the housing facility.

### Animal surgery, 6-OHDA injection, and DP treatments

Rats were randomly assigned (simple randomization) into three groups: sham injury group (surgery and saline injection), 6-OHDA group (surgery and 6-OHDA injection), and 6-OHDA+DP group (surgery, 6-OHDA injection and DP application). For surgery, animals were anaesthetized by intraperitoneal injection of ketamine (100 mg/kg) and xylazine (10 mg/kg) mixture, and placed in a Kopf stereotaxic frame. With the head held firmly in place by the frame, a 2-cm mid-sagittal skin incision was made on the scalp to expose the skull. A dermal drill was used to drill a hole in the skull to expose the dura mater. For 6-OHDA treatment (Sigma-Aldrich, Cas No. 28094–15-7 (year 2017)), a solution of the toxin (8 μg/2 μL) was injected into the substantia nigra at the left side (AP: − 5.4 mm, ML: − 3 mm, and DV: − 8.2 mm from bregma) using a 10-μL Hamilton syringe at a rate of 1 μl/min for a 4-min duration. The sham-operated animals received vehicle (saline) injection. Following the infusion of 6-OHDA or vehicle, the infusion needle was kept in place for 5 min and then slowly withdrawn, and the skin incision closed with stainless steel wound clips. DP (Alfa Aesar, Cas No. 59–52-9) was dissolved in saline and administered to the lesioned animals by daily intraperitoneal injection at 5 mg/kg for 5 weeks, starting immediately (within 5 min) after 6-OHDA injection, and the same dose of saline was administered daily in the same way to the sham group and 6-OHDA group for 5 weeks.

### Open field test

At the end of every week (on days 7, 14, 21, 27 and 35, in the morning) after injury, an animal was placed in a Plexiglas activity box (100 cm × 100 cm × 20 cm) in a dark room. A red light and a camera were placed on top of the box to record the activity of the animal. The activity was recorded for 15 min. The box was thoroughly cleaned with 70% ethanol and water between each animal to avoid thigmotaxic behavior of the animals. The area covered and the distance travelled by each animal were quantified using an automated video tracking system (ANY-maze) to obtain the motor behavioral parameters.

### Rotarod test

The rotarod test, in which animals must balance on a rotating drum, is widely used to assess motor coordination and deficits in neurodegenerative diseases in rodents [41]. The rotarod test was performed weekly, on days 7, 14, 21, 27, and 35 (in the afternoon) after the surgery. The duration that an animal stays on the rod was recorded as a function of drum rotation speed. Animals were first allowed to remain stationary for 10 s. Then the drum began to rotate at the lowest speed, and accelerated to reach the highest speed in 5 min. The time the rat was able to remain on the rotating rod was recorded. The trial was ended if the rat completely fell off from the rungs, or gripped the device and rotated twice without actual walking on the rungs. Each animal received 4 to 7 training sessions before surgery to ensure that it did not fall off and remained there for 30 s after the drum reached the highest speed.

### Mechanical allodynia

The foot withdrawal threshold to mechanical stimuli was tested as an indicator of mechanical hyper-reflexia, according to procedures described in our previous method [20, 34–36, 38]. The test was performed weekly, on days 8, 16, 22, 29, and 35 (in the morning) after 6-OHDA injection. Rats were placed on a metal mesh floor, covered by a transparent plastic box, and allowed to acclimate separately for 10 min in a quiet circumstance before testing. Subsequently, a series of calibrated Von Frey filaments (range: 0.4 g, 0.6 g, 1.0 g, 2.0 g, 4.0 g, 6.0 g, 8.0 g and 15.0 g) (Stoelting, Wood Dale, IL) was applied perpendicular to the plantar surface of the hind-limb with sufficient bending force for 3–5 s. A rapid withdrawal of the hind-limb with or without licking and biting was recorded as a positive response. When a positive response was observed, a lower-grade filament was then applied, and in the absence of a response, the next greater filament was presented. Rats were given at least 1 min for rest between every two stimuli. The up–down method was used to calculate the threshold of mechanical pain and the average score from two hind-limbs was calculated as the final score [42]. The baseline values before brain injury were also recorded for each rat.

### Cold hypersensitivity

The sensitivity to cold application was measured using the 100% acetone-evoked evaporative cooling test, and performed after each of the mechanical hyper-reflexia test. Similar to the mechanical hyper-reflexia test, animals were placed on a metal mesh floor, confined within a transparent plastic box, and acclimated to their surroundings for 10 min before testing. Acetone (0.05 ml) was applied 2 mm from the plantar surface of the hind paw, and the hind paw withdrawal or hind paw licking response indicated cold hyperreflexia. The acetone was applied five times to each plantar paw at intervals of 5 min. Measurements taken before the 6-OHDA injection were used as the baseline values.

### Plantar test

Thermal hyperalgesia was also tested by the plantar test (Hargreaves Apparatus) on days 8, 16, 22, 29, and 35 (in the afternoon) after 6-OHDA injection. Rats were placed into a compartment enclosure on a glass surface. The temperature was set at 52 °C. A mobile heat source was then positioned under the plantar surface of the hind paw and activated with a light beam. The digital timer automatically recorded the latency of paw withdrawal to the nearest 0.1 s. A cutoff time was set at 25 s to prevent tissue damage in the absence of a response. The mean withdrawal latencies for both left and right hind paws were determined from the average of three trials separated by a 5-min interval to prevent thermal sensitization. Paw withdrawals due to locomotion or weight shifting were not counted and the trials repeated.

All the motor and sensory behavioral tests were carried out by an experimenter blinded to the treatment.

### Tissue collection

Animals were deeply anaesthetized with a ketamine/xylazine combination and perfused transcardially with Krebs solution. Three brains from each experimental group were collected and stored in liquid nitrogen until use for Western blotting. The remaining brains (*n* = 5 in each group) were fixed in 4% paraformaldehyde for at least 3 days.

### Immunohistochemistry

Brain tissues fixed in 4% paraformaldehyde were cryoprotected with 15%–30% sucrose, frozen with optimal cutting temperature (OCT) compound, and then cut into 25-μm sections on a cryostat and stored in PBS containing 0.1% sodium azide at 4 °C until use. Serial coronal sections were made at the rostral-to-caudal direction. Sections of both the striatum and SN were processed for anti-tyrosine hydroxylase (TH) immunohistochemistry staining. The sections were washed in 0.01 M phosphate buffer, and then placed in 3% H_2_O_2_/water to quench endogenous peroxidase activity. After washing three times in PBS, the sections were incubated in 5% normal goat serum for 30 min at room temperature, and reacted with anti-TH antibody (1:1 000; Biolegend, San Diego, CA; RRID: AB 2564816) overnight at 4 °C. After several washing steps, the sections were incubated with anti-mouse antiserum IgG for 2 h at room temperature, and then processed using an ABC kit (1:100; Vector, Burlingame, CA; Cat. No. PK-4000) with one drop of solution A and one drop of solution B in 5 ml of PBS for 30 min. After three washes, the sections were incubated in peroxidase substrate solution until the development of desired stain intensity and mounted onto slides. Some sections were processed to control for either monoclonal antiserum or antibody stain. Meanwhile, for anti-acrolein (1:1 000; Abcam, Cambridge, MA; Cat. No. ab48501) immunofluorescence staining, sections of the striatum and SN were used, while for TRPA1 (1:1 000; Novus, Littleton, CO; Cat. No. NB110–40763) immunofluorescence staining, sections of cortex, striatum and SN were used. Standard immunofluorescence staining protocol was carried out as previously described [12]. The number of TH^+^ cells in the SN was counted by Image J, and expressed as a percentage of the control. The immunostaining of acrolein and TRPA1 was quantified with densitometric analysis, and expressed as a percentage of the control. The TRPA1 staining by immunocytochemistry was carried out in SK-N-SH cells, treated with acrolein in the presence or the absence of DP.

### Western blot

Standard Western blot procedures were carried out with the following antibodies: anti-acrolein (1:1 000; Abcam, Cambridge, MA; Cat. No.) and anti-α-syn (1:500; Abcam, Cat. No. ab27766). Briefly, brain tissues were sonicated in 1x RIPA buffer with protease inhibitor. After centrifugation, the supernatant was collected for Western blotting. Sixty micrograms of protein with 20% SDS, β-mercaptoethanol, and 2x Laemmli buffer were loaded onto a 15% Tris-HCL gel and electrophoresed at 80 V for 2–3 h. The proteins were then transferred to a nitrocellulose membrane by electro-blotting at 70 V for 1–2 h (depending on the protein size) at 4 °C in 1x transfer buffer with 20% methanol. The membrane was blocked in 1x casein (Vector) at room temperature for 1 h, and immunolabeled with the primary antibody overnight at 4 °C. The membrane was further incubated with biotinylated anti-mouse or anti-rabbit secondary antibody (Vector) at room temperature for 1 h. The DuoLux substrate (Vector) immunodetection kit was used for chemiluminescent signal, and imaged using a Western blot imager (Azure Biosystems, Dublin, CA). The AlphaView software (Protein Simple, San Jose, CA) was used to quantify the relative signals for each band. Data were normalized with actin and are expressed as the percent of control.

### Cell culture

The SK-N-SH dopaminergic cell line purchased from ATCC (Manassas, VA) was routinely cultured in DMEM (Gibco, Gaithersburg, MD; Cat. No.12430054) supplemented with 10% FBS and 1% penicillin/streptomycin, and maintained at 37 °C in a humidified atmosphere of 5% CO_2_. Twenty-four hours after plating or when the cell density reached 60%–70%, the cells were treated with different concentrations (0, 10, 25, 50, 100, 200, 500, and 1000 μM) of acrolein for 4 h, followed 1 min later by treatment with different concentrations (0, 10, 50, 100, 200 and 500 μM) of DP if necessary.

### Cell viability assay

The SK-N-SH cells were plated in 96-well plates. After various treatments, cell viability was measured by the 3-[4,5-dimethylthiazol-2-yl]-2,5-diphenyl tetrazolium bromide (MTT) method. Briefly, MTT (Sigma, St. Louis, MO; Cas. No.57360–67-9) solution reconstituted in DMEM (0.5 mg/ml) was added in each well (100 μl), and incubated at 37 °C for 1 h. After removing the medium, an equal volume of dimethyl sulfoxide (Sigma, St. Louis, MO; Cas. No.67–68-5) was added to each well to dissolve the remaining formazan crystals. The absorbance of each well was measured spectrophotometrically (Molecular Devices, Spectra) at 570 nm, and the background absorbance at 660 nm was subtracted from these values. Each experiment was repeated for four times.

### Cell morphological observation

The SK-N-SH cells were plated in 6-well plates. After treatment, the cells were fixed in 4% paraformaldehyde for 10 min. After washing three times in PBS, the cells were stained with eosin solution for 5 min, washed by PBS for three times, stained with haematoxylin solution for another 5 min, and then washed by PBS for three times. Photographic images were captured by an Olympus IX51 microscope.

### Statistical analysis

Statistical analysis was carried out using IBM SPSS Statistics 22. Data are expressed as mean ± SD. For statistical analysis, ANOVA and Tukey’s test were used to compare the data. *P* < 0.05 was considered as statistically significant.

## Results

### DP reduces the increased levels of acrolein and α-syn in the brains of 6-OHDA-injected rats

**Fig. 1:**
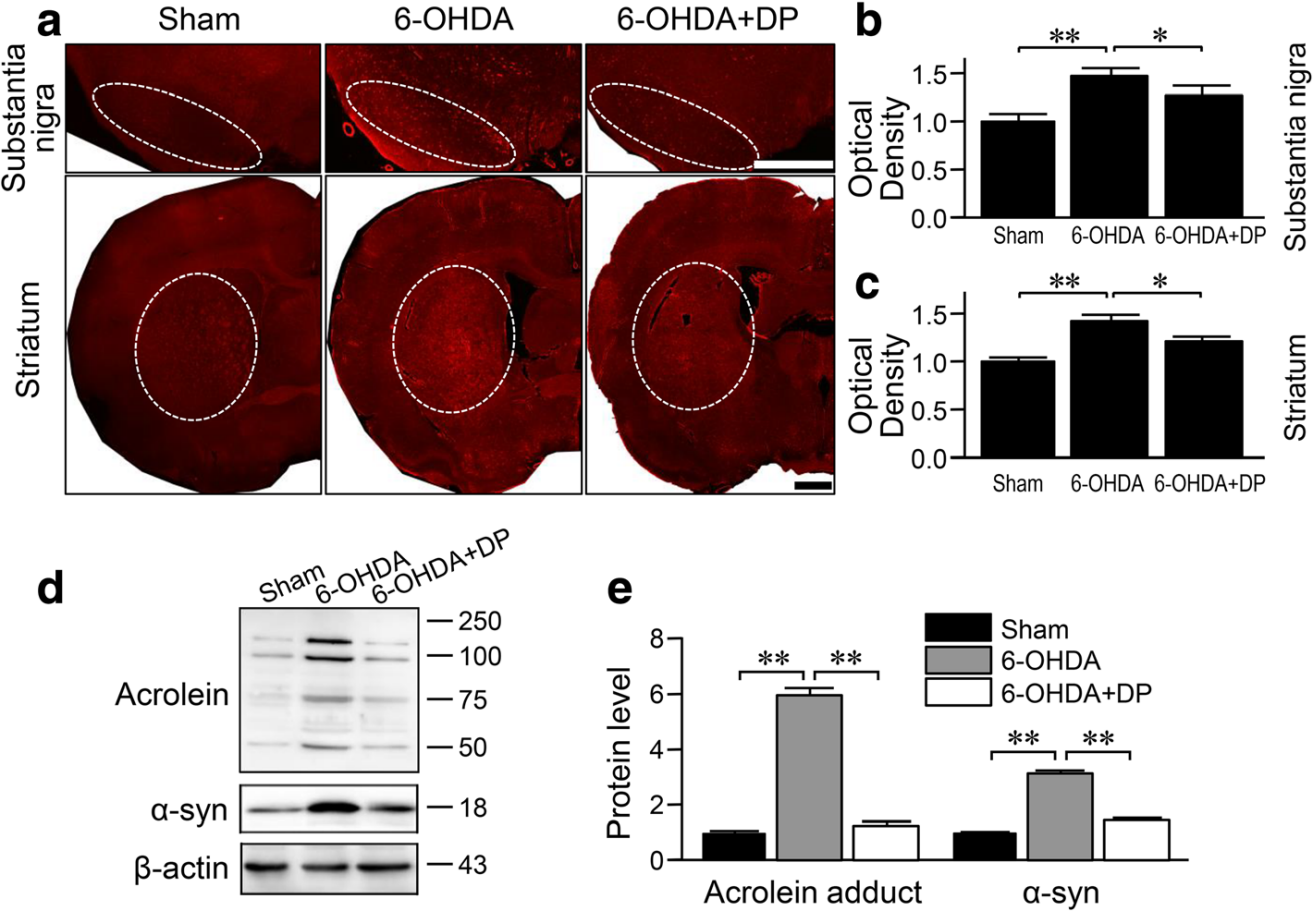
Acrolein and α-syn expression tested by immunofluorescence and Western blot in 6-OHDA rats with DP treatment. **a** Acrolein immunoreactivity was significantly increased in both substantia nigra (SN) and striatum of the 6-OHDA-injected group compared to the sham group when examined 5 weeks post injection. However, this 6-OHDA-mediated increase in acrolein immunoreactivity was alleviated by daily intraperitoneal injection of DP. **b**, **c** Quantitative analysis of acrolein immunoreactivity in **a**. *n* = 5 in each group. **d** Western blots of acrolein and α-syn in the SN. **e** Quantitative analysis of band intensity in **d**. Statistical analysis showed significant increases of both acrolein and α-syn after 6-OHDA toxicity, which were alleviated by DP injection. *P < 0.05, **P < 0.01, ANOVA. Data are expressed as mean ± SD. Scale bar, 1 mm

We have previously found that the injection of 6-OHDA into the medial forebrain bundle significantly increases the level of protein-bound acrolein in the brain [11]. In the present study, anti-acrolein immunofluorescence staining revealed that the injection of 6-OHDA into the SN also significantly increased the level of acrolein in both the SN and striatum of 6-OHDA-injected rats compared to the sham rats, when examined at 5 weeks post-surgery (*P* < 0.01). In addition, the increased levels of acrolein in 6-OHDA rats were significantly alleviated by daily injection of DP (*P* < 0.05) (Fig. 1a–c). Western blotting analysis further showed that both acrolein and α-syn were significantly elevated in the SN of 6-OHDA rats *versus* sham rats, and the elevation was reversed by DP (*P* < 0.01) (Fig. 1e)

### DP reduces the 6-OHDA-mediated DA neuronal death

**Fig. 2:**
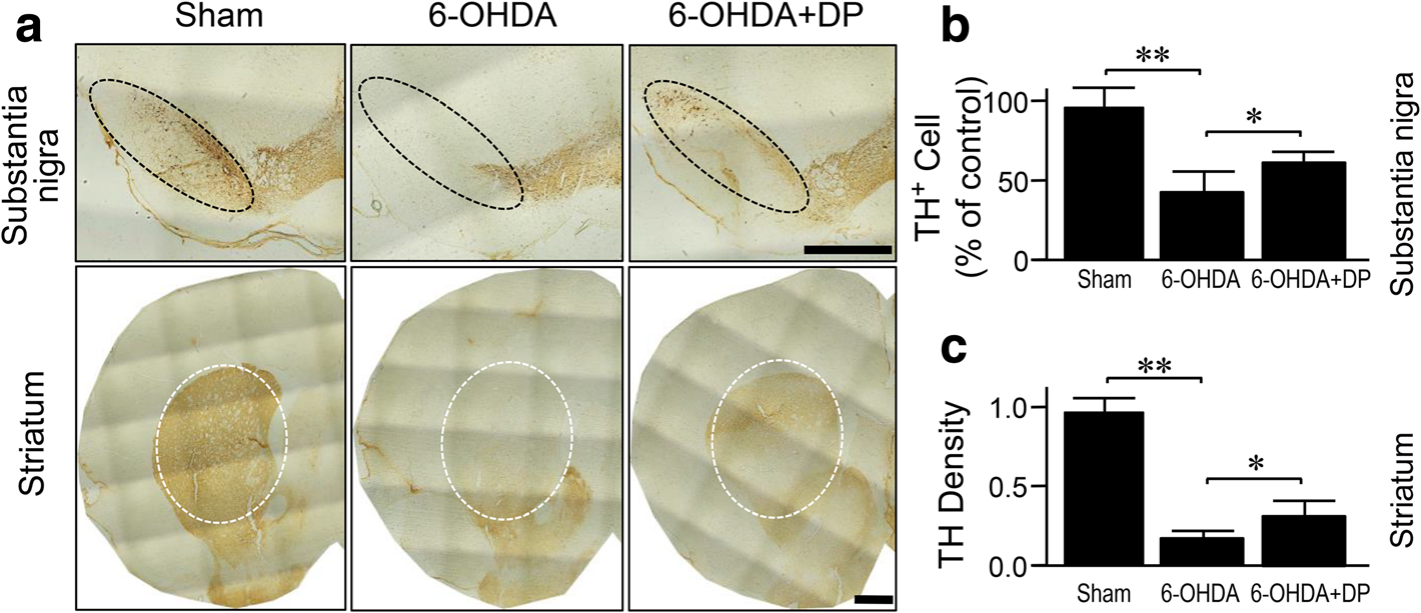
Histological analysis of tyrosine hydroxylase (TH) in 6-OHDA rats with or without DP treatment. **a** TH immunoreactivity was decreased in both substantia nigra and striatum in the 6-OHDA-injected group compared to the sham group when examined 5 weeks post injection. However, the 6-OHDA-mediated reduction in TH immunoreactivity was significantly alleviated by daily intraperitoneal injection of DP. **b, c** Quantitative analysis of TH^+^ cells in the SNpc (**b**) and TH density in the striatum (**c**). *P < 0.05, **P < 0.01, ANOVA. Data are expressed as mean ± SD, *n* = 5 in each group. Scale bar, 1 mm

6-OHDA injections resulted in a 58.3% reduction of TH^+^ cells in the SN (Fig. 2a, b) and a 82.8% reduction of TH immunoreactivity in the striatum compared to the sham injury group, at 5 weeks post-surgery (Fig. 2a, c). Daily intraperitoneal treatment of DP at 5 mg/kg limited the loss of TH^+^ cells to 39.4% in the SN, and the reduction of TH immunoreactivity to 58.5% in the striatum (Fig. 2a–c).

### DP alleviates the 6-OHDA-induced motor deficits

#### Rotarod test

**Fig. 3:**
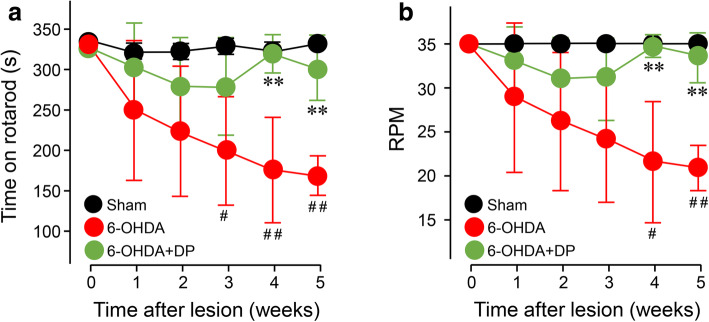
DP mitigates 6-OHDA-induced motor deficits in the rotarod test. The maximum time (**a**) and top speed (**b**) were reduced in the 6-OHDA group, compared with the sham rats. However, DP treatment significantly improved the motor functions at weeks 4 and 5 after injury. DP was applied intraperitoneally at the dosage of 5 mg/kg daily for 5 weeks after injury. **P < 0.01, compared to the 6-OHDA group; ^#^*P* < 0.05, ^##^*P* < 0.01, compared to the sham group; ANOVA. Data are expressed as mean ± SD, *n* = 5 in all groups

The rotarod tests were performed before surgery and at each week after surgery. The maximal time on the rotarod in all the groups (sham injury group, 6-OHDA, and 6-OHDA+DP) before surgery was 330 s. 6-OHDA injection gradually reduced the maximal time on the rotarod, with a significant reduction starting at week 3 (198 ± 67.7 s) compared to the sham injury group (327 ± 5.8 s). However, systemic treatment with 5 mg/kg DP significantly mitigated the reduction at weeks 4 and 5 (318 ± 23.5 s and 300 ± 40.1 s, respectively), compared to the 6-OHDA group at the corresponding time points (174 ± 65.0 s and 167 ± 24.3 s, respectively) (Fig. 3a).

Similar results were obtained when motor ability was measured using the maximal rotation speed that a rat could sustain without falling from the rod. The maximal rotarod rotation speed for rats in all groups before surgery was 35 rpm. 6-OHDA injections resulted in a significant reduction of the maximal speed of the rotarod at weeks 4 and 5 post surgery (22 ± 6.8 rpm and 21 ± 2.6 rpm, respectively), compared to the sham injury group (35 rpm). Similarly, intraperitoneal injections of DP allowed rats to sustain on rotarod at a significantly increased speed at weeks 4 and 5 post injury, when compared with the 6-OHDA group (Fig. 3b).

#### Open field test

**Fig. 4:**
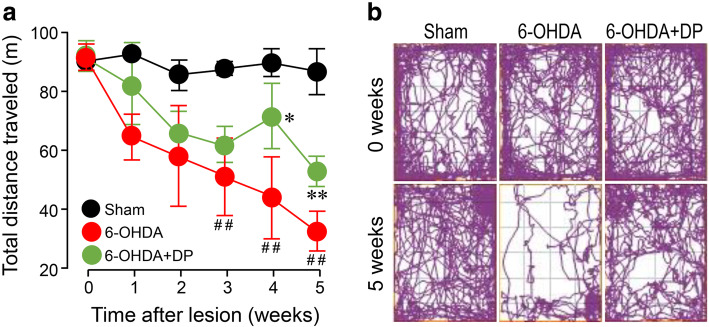
Quantitative motor behavioral analysis based on the open field test. **a** The rats in the sham group walked a greater distance, compared to the 6-OHDA-injected animals. The motor function of the 6-OHDA rats was significantly improved by 5 mg/kg DP. **b** Track plots of the open field test at weeks 0 and 5 after injury. DP was applied intraperitoneally at the dosage of 5 mg/kg daily for 5 weeks after injury. *P < 0.05, **P < 0.01, compared to the 6-OHDA group;    ^##^*P* < 0.01, compared to the sham group; ANOVA. Data are expressed as mean ± SD, *n* = 5 in each group

To measure the general locomotor activity of the rats and their willingness to explore, open field test was carried out before and after surgery (weekly). The 6-OHDA-lesioned rats showed a significant reduction in the total distance travelled beginning from week 3 (51 ± 13.3 m), when compared with the sham group (88 ± 2.2 m). However, systemic treatment with DP significantly increased the motor activity at weeks 4 and 5 (total distances travelled, 72 ± 11.1 m and 53 ± 5.3 m), compared to the 6-OHDA group (44 ± 13.5 m and 33 ± 6.5 m at the corresponding time points) (Fig. 4a). In Fig. 4b, the 6-OHDA-lesioned rats were much less active, and explored a much smaller area, staying mostly in a corner of the box and showing little interest in exploring when compared with the sham rats at 5 weeks post injection. However, DP treatment induced a significant increase in both distance travelled and area explored, compared to the 6-OHDA group (Fig. 4b).

### DP alleviates the acrolein-induced death of SK-N-SH cells

**Fig. 5:**
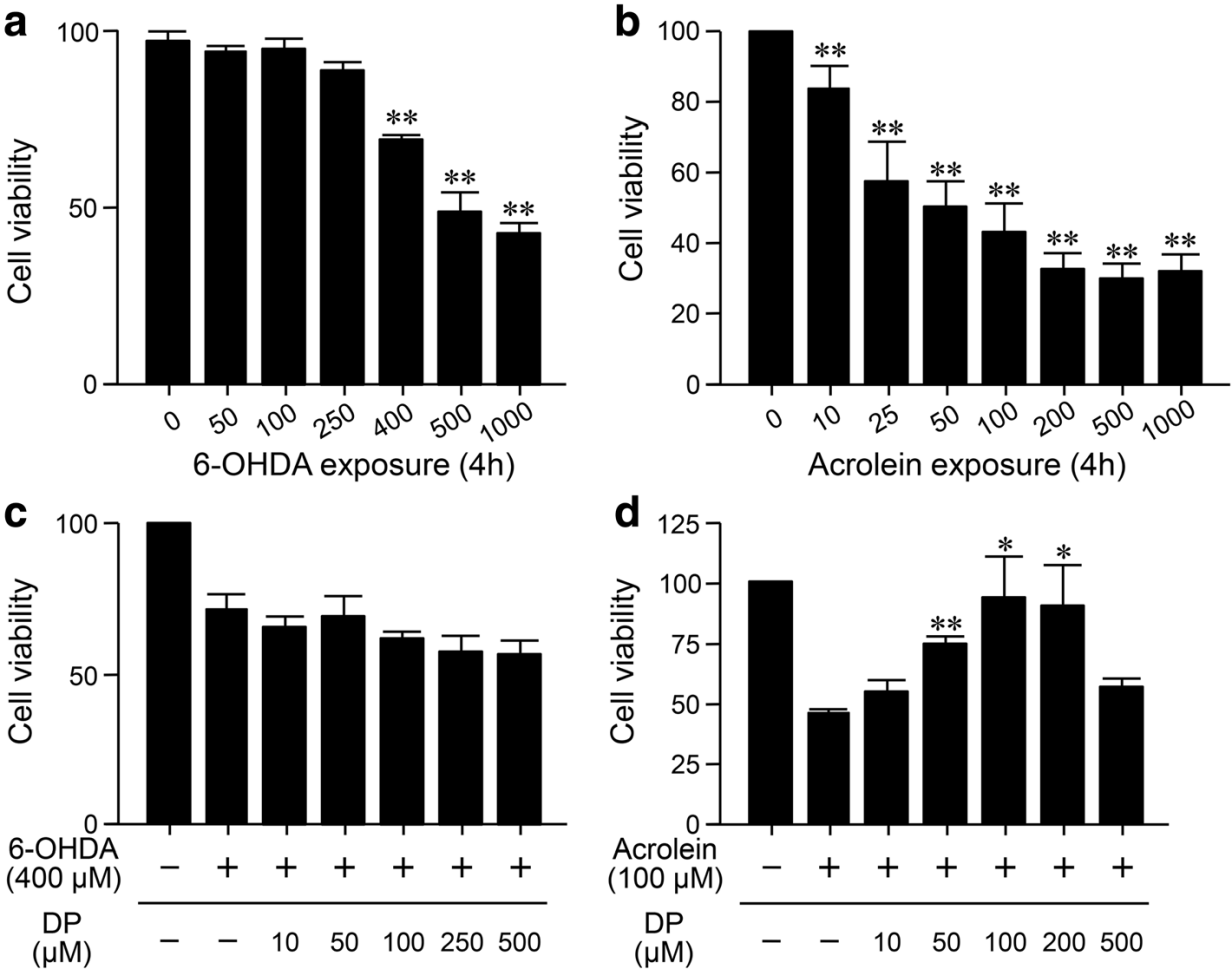
Alleviation of acrolein-induced SK-N-SH cell death by DP. **a, b** SK-N-SH cells were exposed to different concentrations of 6-OHDA or acrolein for 4 h, which induced dose-dependent cell death, based on the MTT test. **P < 0.01, compared to control group. **c** DP (with a 15 min delay following 6-OHDA exposure), at various concentrations did not affect the cell viability, compared to the 6-OHDA (400 μM) exposure group. **d** DP at various concentrations significantly reduced the acrolein-mediated cell death. *P < 0.05, **P < 0.01, compared to the 6-OHDA (**c**) or acrolein only group (**d**). ANOVA and Tukey’s post hoc test. All data are expressed as mean ± SD, *n* = 4 in each group

In vitro experiments were performed to determine if the DP-mediated neuroprotection is a result of acrolein clearance or of 6-OHDA inhibition. First, we evaluated the cytotoxicity of 6-OHDA. Six different concentrations of 6-OHDA were used, ranging from 50 μM to 1 000 μM. The cell viability of the control group (without 6-OHDA treatment) was considered as 100%. The viability of cells exposed to 400 μM 6-OHDA was significantly lower than control (*P* < 0.01; Fig. 5a). However, this cell toxicity was not mitigated by DP at concentrations of 10 μM to 500 μM (Fig. 5c). Next, we evaluated the cytotoxicity of acrolein on SK-N-SH cells. Seven different concentrations of acrolein were used, ranging from 10 μM to 1 000 μM. The viability of cells exposed to acrolein at 10, 25, 50, 100, 200, 500, 1 000 μM for 4 h was 85% ± 6.1%, 57% ± 11.7%, 50% ± 7.5%, 42% ± 8%, 32% ± 4.4%, 30% ± 4%, and 31% ± 4.7% of the control group (no acrolein) (Fig. 5b). In addition, administration of DP at 10 μM, 50 μM, 100 μM and 200 μM, applied 15 min after initial acrolein exposure, increased the cell viability to 55% ± 5.5% (*P* > 0.05), 76% ± 2.8% (*P* < 0.01), 94% ± 17.6% (*P* < 0.05), and 90% ± 17.7% (*P* < 0.05), respectively, compared to the cells with acrolein (100 μM) treatment only (46% ± 2.1%; Fig. 5d). However, with 500 μM DP treatment, the cell viability was only 57% ± 4.2% (*P* > 0.05 *vs* acrolein only), which may be due to the toxicity of DP. The results indicate that DP (50–200 μM) could mitigate the SK-N-SH cell loss after acrolein (100 μM) exposure, but not 6-OHDA.

To further validate the effects of acrolein and DP on SK-N-SH cells, we performed hematoxylin and eosin staining to observe the general morphology of cells. No widespread morphological changes were seen after cell exposure to DP (Fig. S1). However, acrolein treatment for 4 h resulted in gross morphological changes indicative of cytotoxicity. Specifically, the acrolein-exposed cells exhibited nuclear and cytoplasmic shrinkage, accompanied by loss of adhesion. Consistent with the cell viability result  (Fig. 5), DP treatment seemed to alleviate the gross morphological changes resulting from acrolein exposure (Fig. S1).

### DP alleviates mechanical hyperreflexia in 6-OHDA-injected rats

**Fig. 6:**
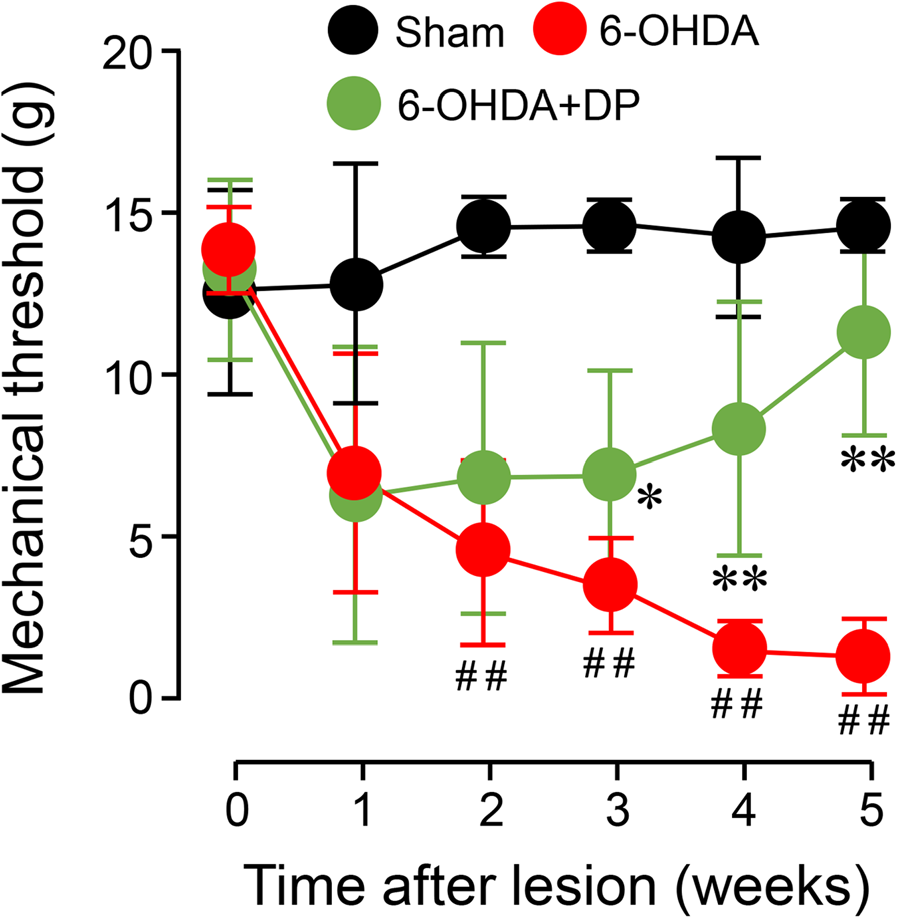
Mitigation of 6-OHDA-induced mechanical hyperreflexia by DP. Within 5 min post-surgery, animals were treated with either 5 mg/kg DP or an equal volume of saline through daily intraperitoneal injection for 5 weeks. Note that beginning at the second week, the 6-OHDA group showed significant mechanical hyperreflexia when compared to the sham group. The mechanical hyperreflexia was significantly attenuated by DP treatment from weeks 3 to 5 post-surgery. *P < 0.05, **P < 0.01, compared to the 6-OHDA group. ^##^*P* < 0.01, compared to the sham group. ANOVA. Data are expressed as mean ± SD. *n* = 5 in each group

Next, we investigated whether the 6-OHDA-lesioned rats displayed sensory hypersensitivity, as acrolein has been linked to neuropathic pain in rodents [32, 43]. As shown in Fig. 6, a significant reduction in the threshold of mechanical hind paw withdrawal was observed in the 6-OHDA group, starting at week 2 (5 ± 2.9 g *vs* 15 ± 0.9 g, *P* < 0.01) and persisting during weeks 3–5 (*P* < 0.01) post 6-OHDA injection, when compared to the sham injury group. Treatment with DP significantly attenuated the 6-OHDA-induced reduction of mechanical threshold at weeks 3, 4, and 5 post 6-OHDA injury (*P* < 0.05 or *P* < 0.01).

### DP alleviates the acrolein-induced thermal hyperalgesia in 6-OHDA injected rats

**Fig. 7:**
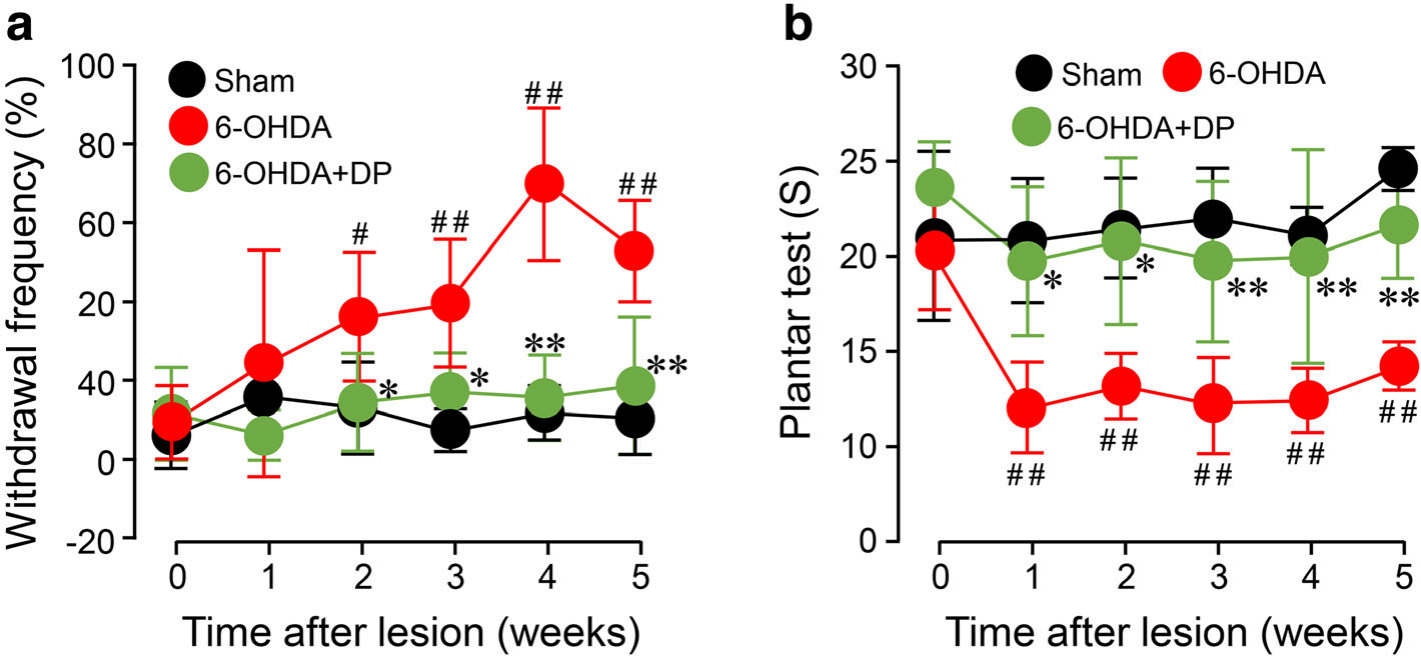
Alleviation of 6-OHDA-induced thermal hyperalgesia by DP. Within 5 min post-surgery, animals were treated with either 5 mg/kg DP or an equal volume of saline through daily intraperitoneal injection for 5 weeks. **a** Animals were tested for thermal hyperalgesia by assessing paw withdrawal frequency (%) using the acetone-evoked evaporation cooling test every week. A significant reduction in the frequency of paw withdrawal was observed after treatment with 5 mg/kg of DP from weeks 2 to 5 post-injury. **b** Animals were also tested for thermal hyperalgesia by assessing the paw withdrawal latency using the plantar test (Hargreaves Apparatus) at 52 °C every week. A significant increase in the paw withdrawal latency was observed after treatment with 5 mg/kg of DP from weeks 1 to 5 post-injury. *P < 0.05, **P < 0.01, compared to the 6-OHDA group. ^#^*P* < 0.05, ^##^*P* < 0.01, compared to the sham group. ANOVA. Data are expressed as mean ± SD. *n* = 5 in each group

The neuropathic pain-like behavior of 6-OHDA-injected rats was also assessed using the acetone spray assay (cold sensitivity) and the plantar test (heat sensitivity). The results showed a significant increase in the paw withdrawal frequency elicited by acetone in the 6-OHDA group, which first emerged at week 2 post 6-OHDA injection (37% ± 16.2%* vs* 14% ± 11.4%, *P* < 0.05), and persisted during weeks 3–5 post injury, when compared to the sham injury group (*P* < 0.05 or *P* < 0.01) (Fig. 7a). However, systemic treatment with DP significantly lowered the 6-OHDA-induced elevated withdrawal frequency at weeks 2, 3, 4, and 5 (*P* < 0.05 or *P* < 0.01).

Similar analgesic effects of DP were also noted in the plantar test. Specifically, a significant reduction in the paw withdrawal latency was observed in the 6-OHDA group, starting at week 1 (12% ± 2.4% *vs* 18% ± 4.3%, *P* < 0.05) and persisting during weeks 2–5 post 6-OHDA injection (*P* < 0.01), when compared to the sham injury control group. Again, systemic treatment with DP significantly mitigated the 6-OHDA-induced reduction of paw withdrawal latency at 1, 2, 3, 4, and 5 weeks post 6-OHDA injury (*P* < 0.05 or *P* < 0.01) (Fig. 7b).

### DP suppresses the acrolein-mediated increase of TRPA1 in both 6-OHDA-injected rats and SK-N-SH cells

We have previously shown that acrolein can stimulate the expression of TRPA1 [37]. Since acrolein is known to be elevated in the striatum, SN and possibly other brain regions in 6-OHDA rats, TRPA1 might also be increased in these regions, potentially playing a pathological role in multiple organ systems (i.e. the nervous system). As such, we set out to examine the possible elevation of TRPA1 in 6-OHDA rats.

**Fig. 8:**
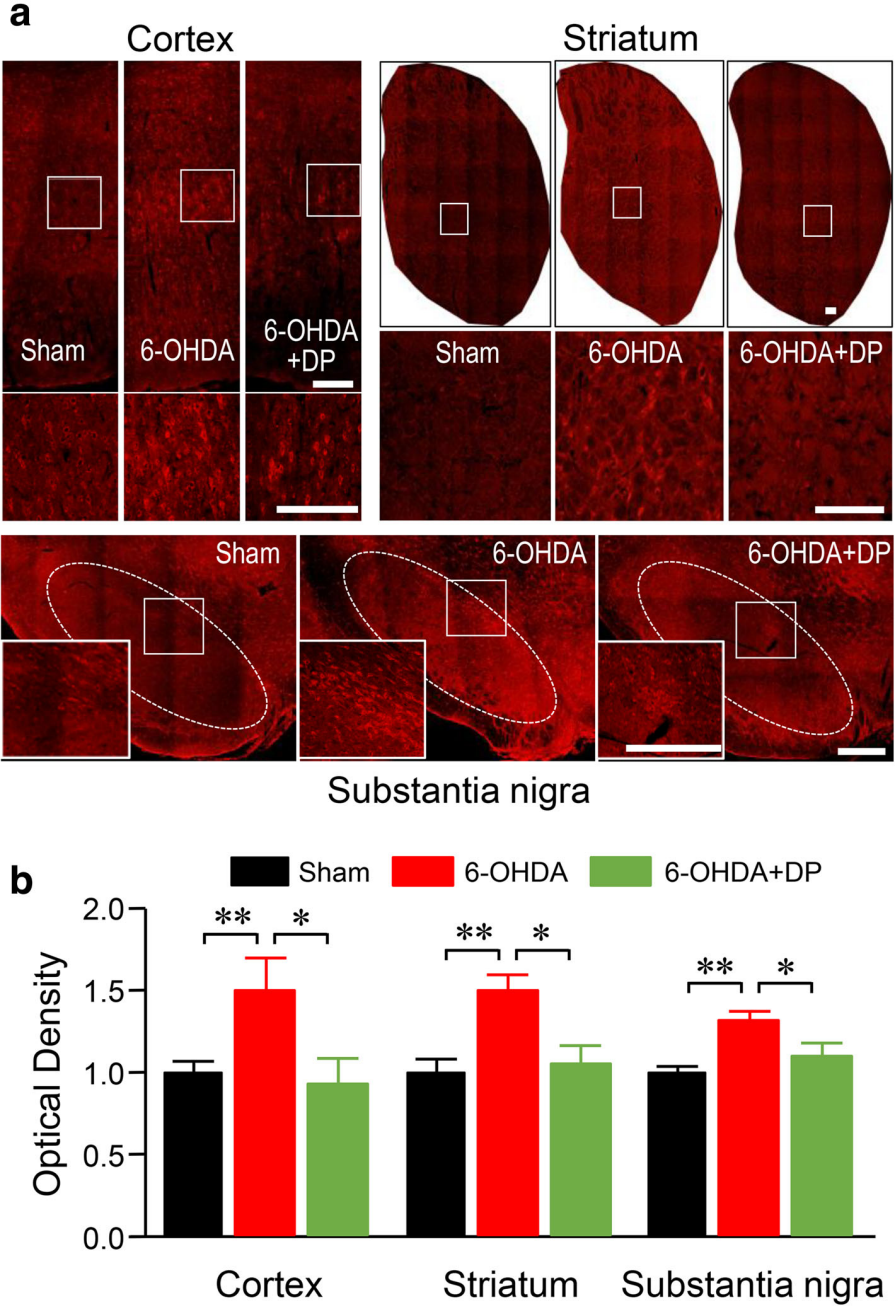
Immunofluorescence images of TRPA1 staining in rats. **a** TRPA1 immunoreactivity was increased in the sensory cortex, the substantia nigra and the striatum in the 6-OHDA-injected group compared to the sham group. However, this increase was alleviated by DP. Images in the lower row or inset represent the enlargement of the boxed areas. **b** Quantitative analysis of optic density values of TRPA1 staining. *P < 0.05, **P < 0.01. ANOVA. Data are expressed as mean ± SD, *n* = 5 in each group. Scale bars, 500 μm

While modest levels of TRPA1 were detected in the sensory cortex, striatum, and SN of the sham injury group, significantly intensified labeling of TRPA1 was detected in the three regions in the 6-OHDA group (Fig. 8a). Optical density (OD) quantification revealed that the values for TRPA1 in the sensory cortex, striatum, and SN of the 6-OHDA group normalized to those of the sham injury group were 1.54 ± 0.19, 1.45 ± 0.05, and 1.34 ± 0.03, respectively (*P* < 0.01 *vs* the sham group). However, DP treatment significantly attenuated TRPA1 intensity in all of these regions in the 6-OHDA rats (0.92 ± 0.16, 1.05 ± 0.12, and 1.11 ± 0.07, *P* < 0.05 *vs* the 6-OHDA group).

**Fig. 9:**
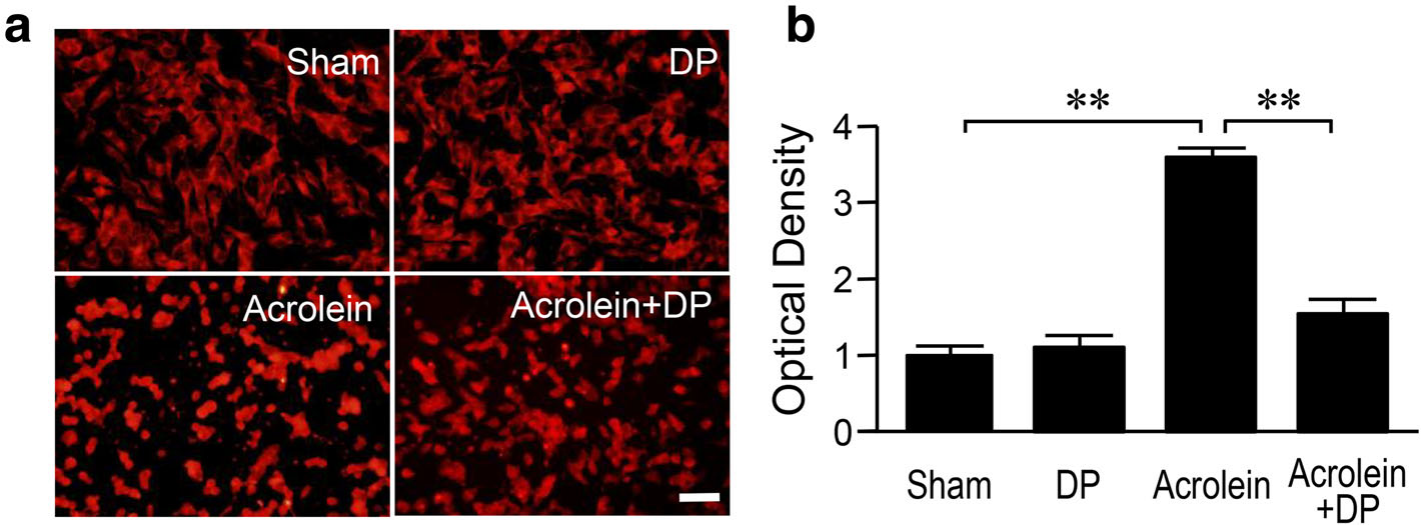
Immunofluorescence staining of TRPA1 in SK-N-SH cells. **a** SK-N-SH cells were exposed to 100 μM acrolein for 4 h, with or without additional treatment with 100 μM DP (with a 15-min delay following acrolein exposure). **b** Quantitative analysis showed that 100 μM DP significantly reduced the acrolein-induced increase of TRPA1. **P < 0.01. ANOVA. Data are expressed as mean ± SD. *n* = 4 in each group. Scale bar, 50 μm

Next, we set out to test the hypothesis that the elevation of TRPA1 in 6-OHDA rats, particularly in DA neurons, is mediated by acrolein. We tested this hypothesis by examining the ability of acrolein to directly stimulate the expression of TRPA1 in DA neurons. Specifically, we examined the change of TRPA1 by directly exposing the SK-N-SH cells to acrolein in a tissue culture system. Results showed significant upregulation of TRPA1 immunostaining in the acrolein-treated cells as compared to controls. Quantitative analysis based on OD revealed that the OD value of TRPA1 in the acrolein-treated cells normalized to that of the sham group was 3.59 ± 0.1 (*P* < 0.01 *vs* sham control). Interestingly, a delayed application (15 min after the onset of acrolein exposure) of DP (100 μM) attenuated the acrolein-induced TRPA1 upregulation (*P* < 0.01; Fig. 9a, b), while the application of DP alone did not change the TRPA1 immunostaining as compared to controls (Fig. 9a, b).

In addition, we compared the body weight of rats between the 6-OHDA group and 6-OHDA+DP group over a 5-week period. Specifically, the two groups of rats (*n* = 5 in each group) received daily intraperitoneal injections of either DP (6-OHDA+DP group) or an equal volume of saline (6-OHDA group) for 5 weeks. As shown in Fig. S2, there was no statistically significant difference in the body weight of rats between the two groups, at any week during the experiments (*P* > 0.05).

## Discussion

In the present study, we showed that DP, a recently identified acrolein scavenger and FDA-approved drug [29], could effectively lower acrolein in the rat brain following 6-OHDA injection, when applied intraperitoneally. This is consistent with our previous report that DP, applied also in the intraperitoneal route, can effectively suppress acrolein elevation following SCI [29]. In addition, the DP-mediated suppression of acrolein was associated with a significant reduction of DA neuronal loss in both the striatum and SN of 6-OHDA rats. Consistent with neuronal tissue preservation, we also found that DP provided motor and sensory functional benefits in this model, including improvement in motor function in the open field and the rotarod tests, and the alleviation of mechanical and thermal sensory hypersensitivity in 6-OHDA rats. Consistent with the in vivo evidence of neuroprotection, DP protected against the acrolein-induced cell death in SK-N-SH cells, in a dose-dependent manner. In addition to the elevated levels of acrolein, we detected a significant elevation of TRPA1 in the brains of 6-OHDA rats. Interestingly, the 6-OHDA-induced TRPA1 upregulation, detected not only in the basal ganglia but also in the sensory cortex, was mitigated with the application of DP, raising the possibility that the TRPA1 upregulation is mediated by acrolein. This possibility was further examined and largely confirmed using in vitro experimentation where direct acrolein incubation led to TRPA1 expression in SK-N-SH cells, which was mitigated by co-application of DP. Taken together, we conclude that DP, a known effective acrolein scavenger, can effectively lower acrolein and mitigate related neurodegeneration in this animal model of PD, resulting in functional benefits for both motor and sensory systems. In addition, acrolein in the 6-OHDA rats may also be responsible for the upregulation of TRPA1 in the brain, which is supported by our in vitro data.

While the 6-OHDA rat model has been widely used to investigate motor and biochemical dysfunctions in PD [44, 45], cautions need to be taken considering the limitations of this animal model. The 6-OHDA rat model is produced by injecting 6-OHDA into the brain, which damages DA neurons through oxidative stress and mitochondrial failure, mimicking an early-to-mid stage of PD, but not late stages of PD (associated with Lewy body formation) [46]. Therefore, the results of this study may help us understand mainly the early-to-mid stage of PD, but the value to the late stage of PD is uncertain.

In our previous report, we have presented evidence that HZ, another known acrolein scavenger, can offer neuroprotection in 6-OHDA rats, as well as in DA cell culture [11]. However, while HZ can effectively sequester acrolein, it is also known to lower blood pressure [23–26]. This potential side effect of HZ hinders the use of HZ as an anti-acrolein strategy for PD. DP, unlike HZ, would not significantly affect blood pressure. As both DP and HZ can offer neuroprotection in 6-OHDA rats, it is reasonable that it is the acrolein-scavenging function, rather than other effects that are unique to each compound, contributes to neuroprotection in this animal model of PD.

DP has two thiol groups that can attack the conjugated C=C and C=O groups of acrolein through the 1,4-addition reaction, forming an unstable olefinic alcohol, which is then converted to stable aldehyde. Furthermore, DP can directly attack the carbonyl group of acrolein through 1,2-addition to form the hemithioacetal, as reported by our previous study [29]. Thus, DP may remove aldehydes more effectively given that each molecule of DP can potentially clear 2 molecules of acrolein, opposed to HZ that scavenges acrolein at a 1:1 ratio. However, a more direct comparison of DP and HZ is warranted to test this hypothesis.

Interestingly, we noticed a difference of DP-induced behavioral benefits between motor and sensory functions. Specifically, DP mitigated motor dysfunction starting at week 4 post injection of 6-OHDA. However, the sensory deficits were mitigated starting at weeks 1 and 2 following 6-OHDA injection. This suggests that hyperalgesia may be more responsive to DP treatment than motor deficits.

Given that 6-OHDA is a pro-oxidative neurotoxin, it is possible that DP mediated neuroprotection through direct binding and blocking of 6-OHDA toxicity. We examined this possibility using an in vitro preparation. The results indicate that DP mitigated cellular toxicity caused by acrolein but not by 6-OHDA (Fig. 5). Therefore, these data suggest that DP can offer neuroprotection by sequestering acrolein, not by preventing 6-OHDA damage to cells.

Taken together, this study presented data suggesting that acrolein scavenging by DP is an effective strategy to combat PD. This could be significant, as most of the established treatments currently available for PD patients offer symptom relief, but are unable to retard the progression of neurodegeneration in PD [47, 48]. In addition, DP is primarily used as a metal ion chelator to treat arsenic, mercury, gold, and other toxic metal poisoning [27]. In PD, both iron and copper are implicated and can accelerate dopamine oxidation [49, 50]. Therefore, DP may offer neuroprotective benefits beyond acrolein scavenging, making it a uniquely attractive PD treatment, a hypothesis which requires further research.

In the current study, the 6-OHDA rats displayed significant sensory hypersensitivity in the mechanical, heat, and cold sensory tests. This is consistent with previous reports that mechanical and heat hypersensitivity are associated with this model [31, 32, 51]. This is also in agreement with clinical observations that neuropathic pain is an important non-motor symptom, detected in up to 85% of PD patients, and greatly impacting their quality of life [43, 52–54]. Interestingly, we observed that DP significantly alleviated neuropathic pain-like behaviors in all three nociceptive modalities of 6-OHDA rats. Although the detailed mechanisms for sensory hypersensitivity in 6-OHDA are not clear, our study does suggest that acrolein is likely involved in  the sensory dysfunction. This is based on the knowledge that acrolein is an algesic aldehyde, known to be involved in neuropathic pain in rodent SCI [17, 20, 38]. In fact, it has been well established that aldehydes, such as acrolein, cause pain by directly binding and activating TRPA1, which is known to elicit the calcitonin-gene-related peptide-dependent pathways, leading to pain [37, 39, 40, 55]. Aldehydes have also been shown to induce wide-spread pro-nociceptive inflammation, further intensifying pain sensation [18, 56, 57]. In fact, we have shown that an injection of acrolein into the spinal cord of healthy rats leads to sensory hypersensitivity, mirroring that from SCI [37, 38, 57]. Taken together, these data support the notion that acrolein is an important inducer of sensory hypersensitivity in 6-OHDA rats and in PD-related pain, likely through a TRPA1-related mechanism.

However, the analgesic effect of anti-acrolein therapy may not be limited to the reduction of TRPA1 binding and activation, but in part result from its ability to mitigate acrolein-mediated DA cell death and preserve DA function in the basal ganglia. There has been increasing literature documenting the importance of DA function for basal ganglia in influencing pain. For example, a correlation between DA function decrease in the basal ganglia and pain sensation increase has been well documented [58]. Furthermore, deep brain stimulation at the basal ganglia has been shown to mitigate chronic pain in PD patients [59]. Consequently, strategies to reduce acrolein, such as the application of acrolein scavengers, could be a feasible approach for mitigating neuropathic pain and improving quality of life for PD patients.

In the present study, we detected the elevation of TRPA1 in sensory cortex, striatum, and SN in 6-OHDA rats. This is consistent with a previous report of the presence of TRPA1 in the somatosensory cortex [60]. It is likely that such TRPA1 upregulation in the brain is mediated by acrolein, as supported by our in vitro tests that demonstrated TRPA1 elevation *via* acrolein exposure, which could be mitigated through acrolein scavenging. Adding to this, we showed previously that an acrolein injection into the spinal cord of healthy rats could induce TRPA1 expression [37]. Although the pathological significance of TRPA1 in PD pathologies was not further investigated in the current study, and to our knowledge remains uncertain, there is some evidence in support of this possibility. First, since TRPA1 elevation was detected in the sensory cortex, it may be directly related to the elevation of pain sensation in 6-OHDA rats. Second, it has been shown that TRPA1 activation may contribute to the pathogenesis of Alzheimer’s disease in rodent models [61]. In addition to neurons, TRPA1 has also been shown to contribute to cardiac tissue damage following myocardial ischemia-reperfusion injury [62], and bone cell apoptosis in rat chondrocytes [63]. Another piece of evidence supporting a pathologic role of TRPA1 is that TRPV1, another member of the TRP channel family, has been shown to mediate death of DA neurons in the basal ganglia [64]. As such, it is very likely that the augmented TRPA1 expression in the sensory cortex contributes to sensory dysfunction and TRPA1 upregulation in the basal ganglia contributes to the degeneration of DA neurons, which subsequently elicits motor and sensory dysfunction. This suggests that in addition to acrolein scavengers, TRPA1 receptor antagonists may also provide neuroprotection and an analgesic effect, a hypothesis that remains to be tested.

## Conclusion

In summary, both in vitro and in vivo data from this study have further strengthened the hypothesis that acrolein is a key mediator of neurodegeneration in 6-OHDA rats. The acrolein-induced pathology is likely mediated by both TRPA1-dependent and -independent pathways in regions beyond the basal ganglia, affecting both motor and sensory functions. Consistently, our data also show that suppressing acrolein by DP can significantly mitigate tissue damage and motor dysfunction typical of PD. The present study and others have provided a strong argument that anti-acrolein is a novel and feasible strategy to combat neurodegeneration in PD, not only because of its functional significance in preclinical observations, but also because of its potentially accelerated path from bench to bedside. Once established, we predict that such treatment strategy will directly benefit PD patients, and potentially patients with other central nervous system disorders linked to acrolein pathologies such as SCI [16, 17, 38, 65], traumatic brain injury [66, 67], multiple sclerosis [68–70], and Alzheimer’s disease [71–73]. Furthermore, acrolein has been linked to cancer [74–76] and aging [77], and exposure has been reported from pollution [78, 79] and smoking [80, 81], which further expands the potential value of anti-acrolein treatment. Therefore, therapies reducing acrolein-mediated neuropathy could have an extensive impact on human health.

## Supplementary Information

**Additional file 1: Fig. S1**. HE staining revealed that DP mitigated the pathological changes of SK-N-SH cells induced by acrolein.

**Additional file 2: Fig. S2**. The effect of 5-week consecutive systemic application of DP on body weight of 6-OHDA-treated rats.

## Data Availability

The datasets used and/or analysed in the current study are available from the corresponding author on reasonable request.

## Abbreviations

PD: Parkinson’s disease
DA: Dopaminergic
DP: Dimercaprol
HZ: Hydralazine
SN: Substantia nigra
SNpc: Substantia nigra pars compacta
TRPA1: Transient receptor potential ankyrin 1
6-OHDA: 6-Hydroxydopamine
SCI: Spinal cord injury
TH: Tyrosine hydroxylase
MTT: 3-[4,5-Dimethylthiazol-2-yl]-2,5-diphenyl tetrazolium bromide
