# Supplementary figures and images for: Acrolein scavenger dimercaprol offers neuroprotection in an animal model of Parkinson’s disease: implication of acrolein and TRPA1
Source: Transl Neurodegener. 2021 Apr 28;10:13. doi: 10.1186/s40035-021-00239-0 (PMC8080346; doi:10.1186/s40035-021-00239-0)

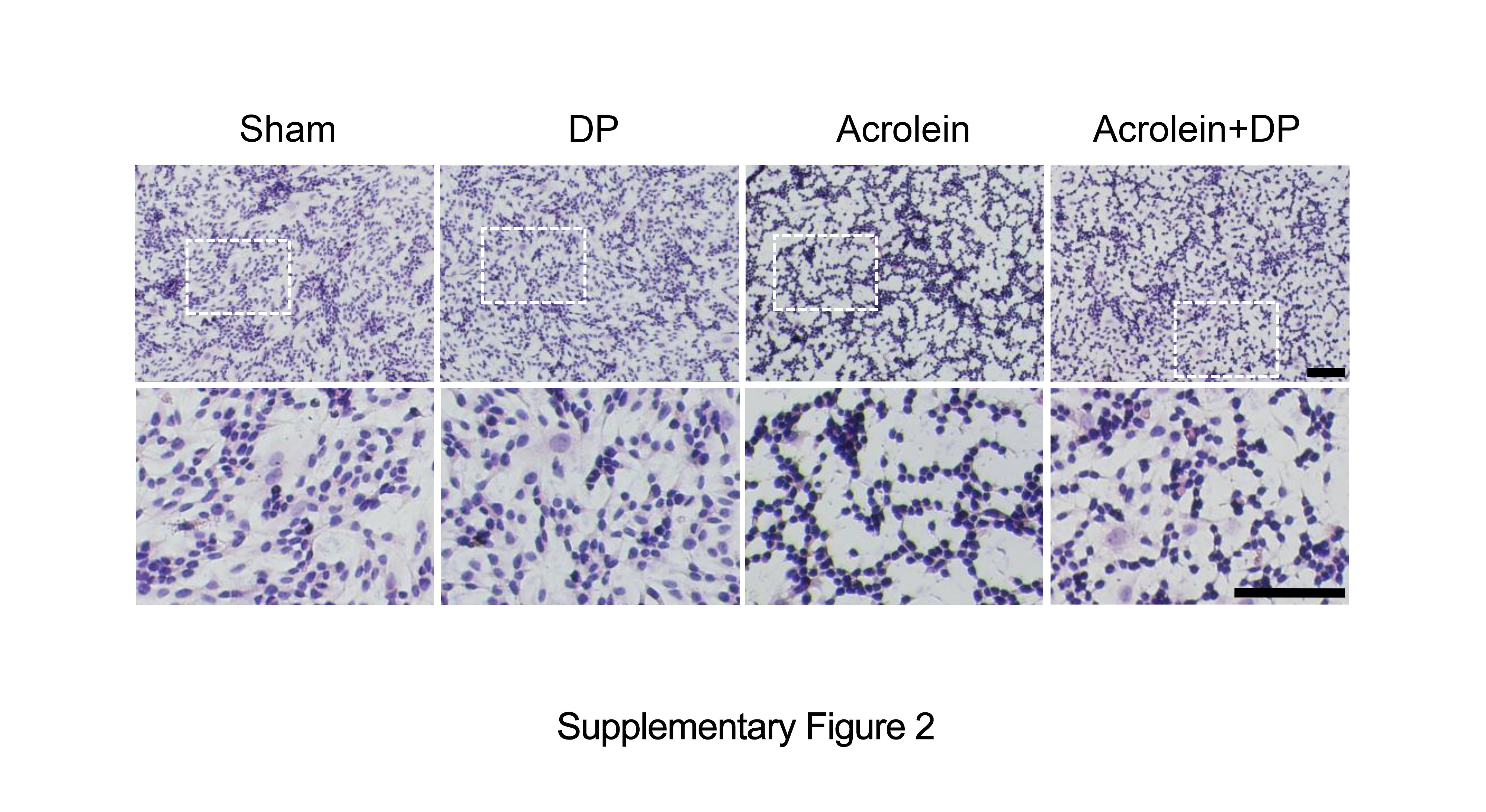

Supplement: Supplementary file 1 — Additional file 1: Fig. S1. HE staining revealed that DP mitigated the pathological changes of SK-N-SH cells induced by acrolein. [file 40035_2021_239_MOESM1_ESM.tif]

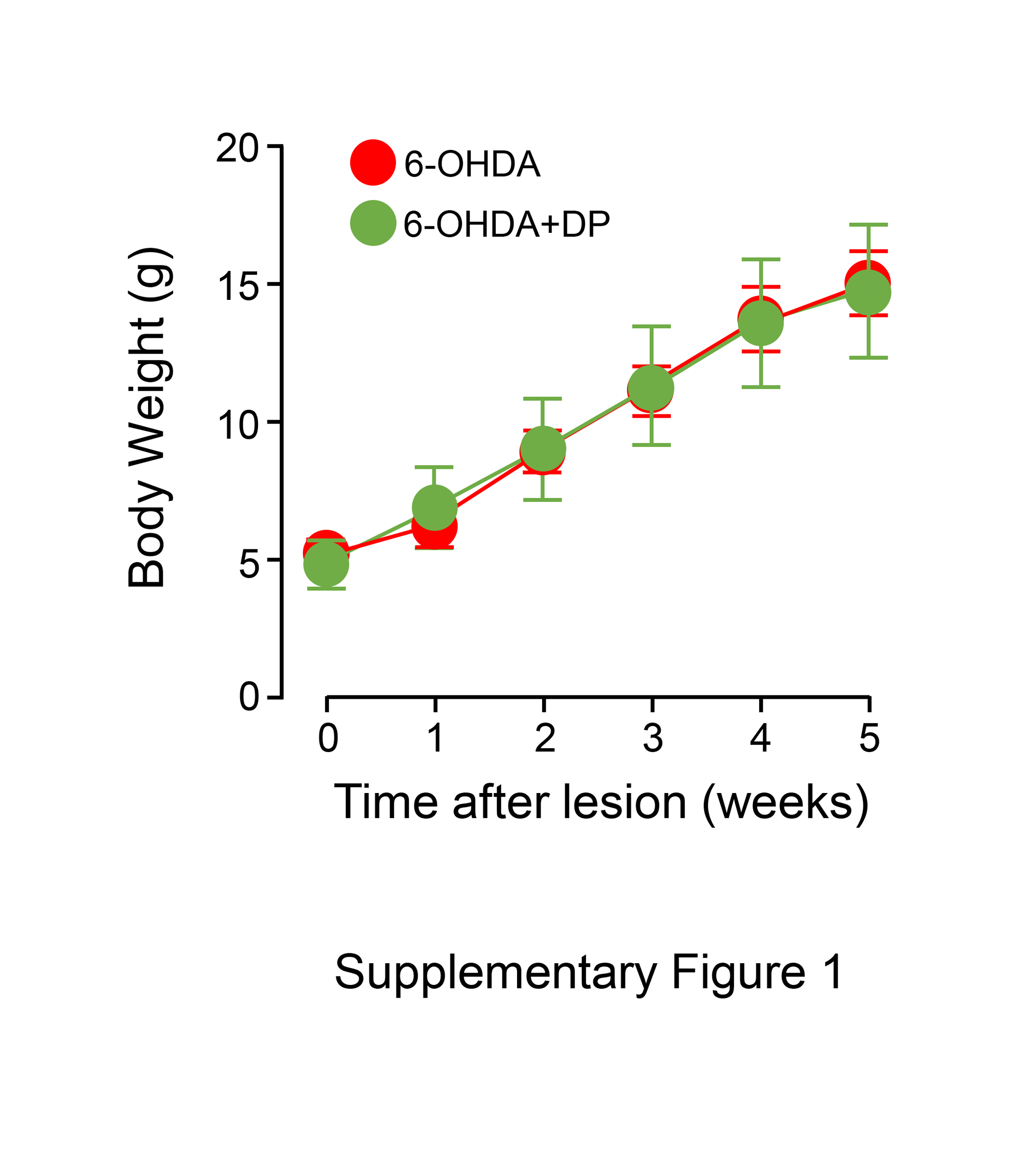

Supplement: Supplementary file 2 — Additional file 2: Fig. S2. The effect of 5-week consecutive systemic application of DP on body weight of 6-OHDA-treated rats. [file 40035_2021_239_MOESM2_ESM.tif]
